# Which career cycle might facilitate students’ development following teachers’ gifted education training?

**DOI:** 10.3389/fpsyg.2026.1813855

**Published:** 2026-04-23

**Authors:** Xiling Qin, Gangling Xiao, Linhai Lv

**Affiliations:** 1 School of Education, Nanjing University, Nanjing, China; 2 Graduate School of International Studies, Seoul National University, Seoul, Republic of Korea; 3 Institute of Higher Education, Fudan University, Shanghai, China; 4 School of Marxism, Shanghai Publishing and Printing College, Shanghai, China

**Keywords:** 21st century skills, gifted education, heterogeneous treatment effects, propensity score stratification, propensity score weighting, teacher training

## Abstract

**Introduction:**

The educational philosophy advocating that “students from all economic and cultural backgrounds should reach their full potential” has not extended to the most capable student cohort, leaving gifted students underserved. Over the past decade, academic attention has turned to the teachers serving these students, as growing evidence indicates that a widespread lack of professional development support for gifted education. This means that even when teachers lack essential knowledge and experience regarding the characteristics and needs of gifted learners, they still bear significant responsibilities.

**Methods:**

This study, grounded in the five-stage theory of teaching careers, examines how teacher training related to gifted education impacts student learning outcomes.

**Results:**

Through propensity score matching and heterogeneity treatment effects, it reveals that the effectiveness of gifted education training follows an M-shaped curve as teacher experience accumulates.

**Discussion:**

This empirically supports the heterogeneous treatment effect (HTE) of teacher training, laying the foundation for personalized professional programs for gifted educators.

## 1 Introduction

Cognitive, interpersonal, and intrapersonal abilities are crucial for positive development and achievement across multiple domains, including education, employment, health, and well-being (OECD, 2019). However, empirical research on the antecedents or associated factors influencing these variables remains urgently needed. Simultaneously, the educational philosophy advocating that “students from all economic and cultural backgrounds should reach their full potential” has not been extended to the gifted student cohort. Just as students struggling academically require supportive interventions, top performers should also receive specialized guidance to ensure they gain opportunities to expand their knowledge and skills.

The standards for gifted educators in the United States are jointly developed by NAGC and CEC. In 2024 (National Association for Gifted Children; Council for Exceptional Children, 2024), the organization updated its initial preparation standards for gifted educators. Compared to the previous 2013 version (National Association for Gifted Children; Council for Exceptional Children, 2013), the latest edition features three prominent characteristics: First, it introduces new requirements for teachers to address students’ social, emotional, and psychological dimensions. This shift reflects a transition in gifted educations focus from “high academic achievement” to “whole-person development,” correcting the misconception that “giftedness” equates to “high scores.” Second, it prioritizes teacher professional development (hereafter referred to as “PD”). NACG and CEC emphasize that PD should be supported by evidence-based practices and facilitated through effective instructional and environmental supports. Third, it maintains the critical importance of recognizing student individuality. Teachers are required to provide tailored instruction that meets student needs, respond to the interests and talents of the gifted and talented, and design meaningful, challenging learning experiences.

CPC Central Committee and the State Council (2025) has issued the “Outline for Building a Leading Education Nation (2024–2035),” which places particular emphasis on building a high-quality, professional teaching workforce and enhancing teachers’ professional competencies. Regarding PD program for teachers, the outline requires strengthening training for all teachers. Additionally, Ministry of Education of the People’s Republic of China (2020) introduced the “Guidelines for Teaching Curricula for Primary and Secondary School Teachers” in 2020. This initiative aims to advance the PD of primary and secondary school teachers while enhancing the relevance and effectiveness of training programs. The guiding principle behind this framework is that teacher training for primary and secondary school teachers should promote their professional growth, with the ultimate goal of fostering student outcomes.

Unlike other countries, teacher supply and attrition are not significant concerns for public school teachers in China’s basic education sector, as they enjoy quite stable salaries and benefits. However, in terms of career development, this job security may actually dampen teachers’ enthusiasm for participating in PD programs, thereby potentially affecting teaching quality.

## 2 Literature review

The scope and content of professional development are broadly defined in the literature. Little (1993) posits that professional development encompasses any activity designed partially or primarily to enhance the classroom practice and educational reform of school staff. Although PD training varies significantly in content and format, it shares “a common purpose” - most often, the improvement of student learning outcomes (Guskey, 2002).

### 2.1 Teacher training related to gifted education

For decades, research on teacher professional development training has primarily focused on two themes. The first involves “antecedent variables” related to teachers, examining which characteristics influence their decision to participate in PD. Examples include teachers’ expert knowledge and self-efficacy (Carney et al., 2016; Chen et al., 2021; Sancar et al., 2021). The second records “outcome variables” related to teachers, such as changes in satisfaction, attitudes, or beliefs following training participation (Guskey, 2002). Existing literature reveals significant research gaps: How do different teachers perform and benefit during professional development? Beyond teacher-related variables, can professional development training tangibly impact student growth? Furthermore, as a key benchmark for evaluating educational investment, is there empirical support for changes in student learning outcomes?

From a methodological perspective, on the one hand, most studies on teacher training employ interviews or open-ended surveys (Avidov-Ungar et al., 2023). On the other hand, quantitative research has measured the effectiveness of such training, though the evidence typically exhibits low power. For instance, by comparing students’ outcomes at the beginning and end of the semester using *t*-tests, it was found that teacher participation in PD activities could enhance student learning (Antoniou and Kyriakides, 2013). Research designs like this are largely incapable of controlling for confounding variables, presenting numerous threats to internal validity. It is difficult to rule out the possibility that observed changes stem from factors unrelated to professional development. In other words, endogeneity issues remain unresolved.

Further study about professional development training for teachers related to gifted education reveals that no work on giftedness, talent, and creativity dedicates an entire chapter or more to teacher training, such as Plucker and Callahan (2021) or Robinson and Kolloff (2021). As noted by the World Council for Gifted and Talented Children (2021), professional development training in gifted education requires stronger empirical support beyond mere rationales. In other words, there exists a crisis of confidence regarding whether training that equips teachers with specialized knowledge and skills can tangibly contribute to the learning and development of gifted students - and specifically, which student competencies such training can enhance.

Unlike general educators, gifted education places higher demands on teachers. On one hand, gifted students possess unique learning needs: beyond advanced knowledge, they tend to actively seek information, generate novel ideas, and engage in complex thinking (Gallagher, 2013). Without appropriate instructional interventions, gifted learners may lose interest in formal education and disengage from learning (Preckel et al., 2010). Furthermore, within the gifted population, as many as one in six students exhibit some form of learning difference alongside their high intelligence (World Council for Gifted and Talented Children, 2021). Consequently, educators must not only address the unique learning and developmental needs of gifted students but also account for the diversity within this cohort. They must help students identify and structure real-world problems aligned with their interests while simultaneously providing the methods, resources, and skills necessary to solve these specific challenges.

However, a review of research on teacher characteristics and instructional practices reveals that a substantial body of evidence indicates that few teachers, whether pre-service or in-service, are capable of providing specialized instruction for high-ability students (Robinson and Kolloff, 2021). This stems from teachers’ lack of knowledge regarding how to adapt curriculum for students whose abilities extend beyond the scope of grade-level courses and prescribed standards documents (Callahan and Reis, 2004; Hertberg-Davis, 2009). Teachers’ self-reports further confirm that insufficient knowledge of gifted education pedagogy leaves them feeling ill-equipped to effectively serve gifted students (Antoun et al., 2020). This underscores the need for the gifted education field to provide teachers with robust training, enabling them to create novel and appropriate learning experiences that support students’ capacity development.

Conversely, if teachers lack professional training, it will negatively impact teaching practices, student outcomes, and the entire education sector. Specifically, in terms of teaching practices, untrained teachers not only lack knowledge about how and when to adjust instructional pace for gifted students but also fail to understand how to employ differentiated instruction strategies to increase the depth and complexity of instructional content (Van Tassel-Baska et al., 2021). Regarding student outcomes, it may result in insufficient challenges, inadequate differentiated instruction, increased underachievement or dropout rates among students, and diminished achievement for all gifted learners (Reis and Renzulli, 2010). For the education sector, teachers’ lack of specialized expertise may lead to misconceptions about identifying and nurturing gifted students, exacerbating the separation between gifted education and general education (Sánchez-Escobedo et al., 2020).

### 2.2 21st century skills

**Figure 1:**
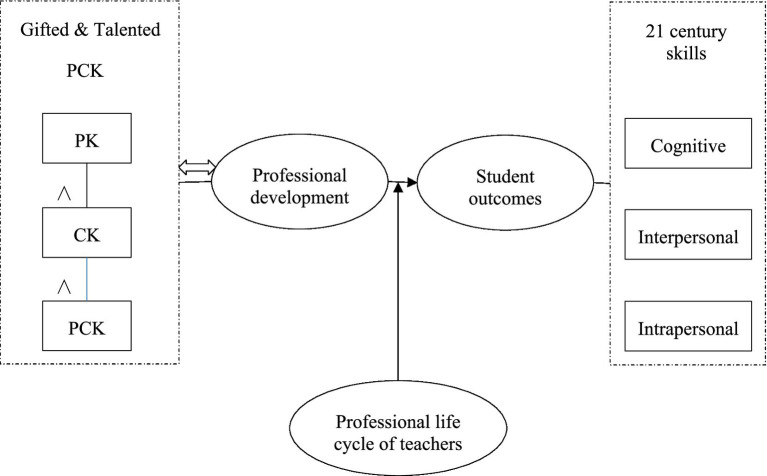
Conceptual Framework. *∧ means logical AND (all conditions must hold simultaneously); ⇔ means if and only if.

The report “Educating for Life and Work: Developing Transferable Knowledge and Skills in the 21st Century” by the National Research Council (2012) distilled three broad domains of competence: cognitive, interpersonal, and intrapersonal skills, collectively referred to as “21st Century Skills”(see Figure 1). These three dimensions of 21st-century skills integrate cognitive, psychosocial, and emotional domains - marking a distinct departure from traditional education’s narrow focus on cognitivism. Cognitivism was limited to examining internal mental processes within individual learners, viewing social interaction merely as a backdrop to learning. The introduction of 21st century skills address the shortcomings of cognitivism by recognizing social interaction as a fundamental factor shaping what individuals can know, do, and become. It views skill development as a constructive process resulting from continuous interaction with biological, psychological, and sociocultural factors. This shift reflects progress toward a more comprehensive, healthy, and productive concept of human development (Pellegrino, 2020).

Academic circles have interpreted the three dimensions of 21st-century skills. First, regarding cognitive ability, numerous renowned frameworks exist, including Carroll’s (1993) three-stratum theory, Snow’s (1984) Radex model, and Sternberg’s (1985) triarchic theory. Second, about interpersonal skills, as the era of lone genius fades (Suresh, 2013), complex challenges and opportunities now demand the efficient integration of diverse thinking styles. Interpersonal acuity and collaborative abilities are becoming increasingly critical. Third, about intrapersonal ability, Gardner (2006) emphasizes the importance of intrapersonal intelligence, which core lies in identifying and evaluating one’s strengths, weaknesses, talents, and interests, and utilizing these insights to develop adaptive, goal-oriented self-direction. In his Differentiated Model of Giftedness and Talent (DMGT), Gagné (2004) highlights that intrapersonal or self-management act as catalysts in talent development, influencing growth through both directional and intensity factors.

The development of the three major ability domains - cognitive, interpersonal, and intrapersonal - may be asynchronous both across different groups and within the same individual. For instance, while gifted students may lead their peers in cognitive ability compared to average students, they do not necessarily exhibit giftedness in all domains (Winebrenner, 2020), and other skills still require gradual cultivation (Gagné, 2004). Similarly, within the gifted population, some students may exhibit exceptional intellectual abilities while lagging in social–emotional development (Silverman, 2012). This implies that gifted educators must not only recognize how the learning styles and socio-psychological needs of gifted students differ from those of their peers, but also hold professional awareness of the internal variations and diversity within the gifted cohort itself.

Both cognitive ability and “non-cognitive ability” - comprising interpersonal and intrapersonal skills - exhibit plasticity. Psychologists emphasize that one should not assume students possessing cognitive ability and possess psychosocial competency, nor should it be assumed these skills emerge without direct instruction and teaching (Subotnik et al., 2011). In other words, the diversity in students’ developmental trajectories demands that educators provide multifaceted perspectives on learning, addressing both cognitive and psychosocial needs (Cathcart, 2020). It is evident that gifted students thrive only when teachers guide them in meeting their cognitive and psychosocial developmental requirements. Thus, the key to improving school education lies with the teachers.

## 3 Theoretical foundations

### 3.1 Pedagogical content knowledge

Shulman (1986) proposed the PCK model for teacher professional development training, emphasizing that such training should encompass pedagogical knowledge, content knowledge, and subject pedagogical knowledge. Schulman’s PCK model aligns with perspectives from other educational experts. For instance, Robinson and Aronica (2009) contends that secondary school teachers require strong preparation in three domains: pedagogical knowledge, subject matter knowledge and subject-specific pedagogy.

In the PCK model, pedagogical knowledge encompasses knowledge related to teaching strategies, classroom management, and other areas. As scholars have pointed out, training for teachers engaged in gifted education must include classroom management skills (Tomlinson and Allan, 2000). Content knowledge relates to the academic discipline itself. Teachers less familiar with a particular subject area can expand their knowledge base by participating in professional development training within that specific field. The overlap between pedagogical knowledge and content knowledge (PCK), which requires teachers not only to be proficient in the subject matter but also to be able to apply appropriate teaching strategies to help students understand.

This study designed a dichotomous independent variable based on Shulman’s PCK framework, examining whether teachers’ PD training experiences encompassed the following three dimensions: teaching subject-specific knowledge to gifted students, involving pedagogical knowledge related to gifted education, and delivering higher-level subject knowledge to gifted students (e.g., teaching university-level subject knowledge to secondary school students). When teachers selected “no” for all three aspects, they were considered to have not participated in training specifically targeting gifted education.

### 3.2 The professional life cycle of teachers

Michael Huberman has made outstanding contributions to research in teacher education, having taught at the University of Geneva in Switzerland and Columbia University in the United States. His co-edited work with Guskey, Professional Development in Education: New Paradigms and Practices, was named “Staff Development Book of the Year” by the National Staff Development Council (NSDC). The “Professional Life Cycle” model stands as one of his most notable achievements. American Educational Research Association (AREA) also established the Michael Huberman Award for Excellence in Outstanding Scholarship on the Lives of Teachers to scholars who have made distinguished contributions to research on teachers’ lives.

The professional life cycle comprises five distinct phases. Starting from the first year a teacher enters the education field, the initial 1 to 3 years are termed the “career entry”; years of 4 to 6 constitute the “stabilization” phase; years of 7 to18 represent either an “activism” or “self-doubts” state; years of 19 to 30 are termed the “serenity” or “conservatism” period; and years of 31 to 40 constitute the “disengagement” phase approaching retirement (Huberman et al., 1993). Detailed characteristics of each stage are outlined in Table 1.

**Table 1:** The professional life cycle of teachers.

Years of teaching	Career cycle	Characteristics
1 ~ 3	Entry	Survival or discovery
4 ~ 6	Stabilization	Clear commitment to the profession, comfortable with teaching environment
7 ~ 18	Active VS. self-doubts	Action-oriented teaching experiments, career advancement opportunities, or crises of professional reassessment
19 ~ 30	Serenity/conservation	Maintaining a rational distance from students, no longer having high expectations of oneself
31 ~ 40	Disengagement	Bitter or serene

As shown in Table 1, once teachers successfully navigate the demanding survival struggles and teaching challenges of the first 3 years, they experience a sense of accomplishment and excitement about discovering new things. They typically enter a stable phase - the second stage - and gradually establish themselves in their professional track. It is not until the third phase - the mid-career stage - that educator may find teaching demands become routine, prompting them to explore new instructional materials and strategies. However, some teachers in this same phase may also encounter fresh challenges and fall into self-doubt. For instance, after reflecting on the gap between actual teaching outcomes and “expected” results, they may begin questioning the meaning and value of their work during the first half of their professional cycle. By the fourth stage, many educators return to the status of calm. Finally, in the twilight of their careers, teachers may feel bitter or serene, lacking the passion to commit further to their work (Huberman and Middlebrooks, 2000).

This study proposes a heterogeneity hypothesis based on the lives of teachers. Specifically, it posits that the effectiveness of teachers’ participation in training related to gifted education varies according to the five career stages outlined by this theory. In other words, teachers at different states derive distinct benefits from professional training, which manifests as varying impacts on student learning outcomes.

## 4 Methods

### 4.1 Key concept

Professional development and teacher training are two ends of the same continuum. Early understanding of teacher training tended to favor short-term, externally driven models focused on the input of knowledge. In contrast, professional development emphasizes the construction and transformation of knowledge and has gradually gained widespread acceptance. Although the two terms are often used interchangeably, professional development represents a long-term, higher-level process of knowledge reconstruction compared to teacher training (Avalos, 2011).

A representative definition of professional development is as follows: Borko (2004) approaches the concept from the perspective of temporal boundaries, arguing that professional development is not a one-time training event, but rather a continuous series of learning opportunities after teachers have obtained their initial certification. Timperley et al. (2007) further emphasize the outcomes-oriented perspective, defining professional development and learning as formal and informal learning experiences designed to development knowledge and skills teacher need to achieve better student learning outcomes.

In the field of gifted education, VanTassel-Baska (2003) applied Shulman’s PCK framework to curriculum planning and instructional design for gifted students, working backward from student outcomes to determine teacher behaviors. In a review study, Rogers (2007) noted that gifted students cannot be fully developed through merely slight modifications to standard classroom instruction; they require instructional arrangements and curricular experiences that align with their abilities, interests, and learning styles. This reflects an approach that integrates content knowledge, pedagogical knowledge, and the learning characteristics of gifted students. Building on this, Weber and Mofield (2023) explicitly expanded PCK into GT-PCK (Gifted and Talented - Pedagogical and Content Knowledge), stating that professional development for teachers of gifted students should encompass deep subject knowledge, specialized instructional strategies, and a profound understanding of the social and emotional needs of high-ability learners.

Based on a review of existing research on teacher training and professional development, this study adopts the following definition: Professional development for teachers in the gifted education field refers to all formal and informal learning opportunities that enable teacher, after obtaining certification, to develop the content knowledge, pedagogical knowledge and deep understanding for the needs of talented students.

### 4.2 Research design

The research question of this study is to examine the impact of secondary school teachers’ participation in gifted education training on student learning outcomes, and how its impact is influenced by the teachers’ career stage. In statistical terms, the independent variable is whether teachers participate in professional development in the field of gifted education; the dependent variable is cognitive, interpersonal, and intrapersonal development of gifted students; and the moderator is the life cycle of teachers. In terms of variable types, the moderator and the dependent variable are both continuous variables, while the independent variable is a dichotomous variable.

In the conceptual framework diagram, the design of the independent variables is based on the PCK model, incorporating the findings of Van Tassel-Baska, Rogers, Weber Mofield, and others who have applied this framework to the field of gifted education. It is summarized by three indicators: pedagogical knowledge (PK), content knowledge (CK) and pedagogical content knowledge (PCK). Only when a teacher selects “Yes” for all three items is that teacher considered to have participated in professional development in the gifted education field; if a teacher selects “No” for at least one item, he or she is considered not to have participated in a professional development program for gifted education. Specifically, the question corresponding to PK is “Does the professional development you participated in include pedagogical knowledge for gifted students?,” the question corresponding to CK is “Does the professional development you participated include subject content knowledge?,” and the question corresponding to PCK is “Does the professional development you participated in include pedagogical knowledge for subject content tailored to gifted students?”

The outcome variable is based on the 21st Century Skills proposed National Research Council, which encompasses three dimensions: cognitive, interpersonal, and intrapersonal. Among these, cognitive domain as involving thinking and related abilities; interpersonal competencies are those used both to express information to others ... and respond appropriately; intrapersonal ability includes self-regulation – the ability to set and achieve one’s goals. To be specific, cognitive includes nonroutine problem solving critical thinking, systems thinking; interpersonal skills include complex communication, social skills, teamwork, cultural sensitivity, dealing with diversity; intrapersonal skills include self-management, time-management, self-development, self-regulation, adaptability, executive functioning (Council N. R, 2012) In addition, many researchers refer to interpersonal ability and intrapersonal ability as social–emotional ability (Schoon, 2021). The dependent variable is operationalized using a questionnaire (IPQ) designed by Hong et al. specifically for teachers of gifted students.

The moderator variable is operationalized within Huberman’s theory and represented by teacher’s years of teaching. To better align the operationalization of the teaching life cycle with the definition in Huberman’s theory, we use years of teaching experience rather than chronological age as the variable.

In terms of analysis, this study employs two statistical methods: Propensity Score Matching (PSM) and the Heterogeneity Treatment Effects (HTE). The advantages of using PSM in this research are as follows: First, teachers exhibit self-selection in deciding whether to participate in training, which introduces significant systematic bias in traditional estimation methods (such as Ordinary Least Squares (OLS)). PSM reduces the impact of such selection bias. Second, it resolves the challenge of establishing a control group in previous teacher professional training studies, which often employed single-group pre-post designs, thereby enhancing the power of causal inference. The HTE employed in this study follows the PSM to further examine potential heterogeneity in intervention effects (Xie, 2013), assuming that the effectiveness of PD training varies across teachers at different career stages.

Following the counterfactual model framework, let $D_{i}=1$ denote participation in training related to gifted education professional development, and let $D_{i}=0$ denote non-participation in such training. Participation is determined based on the following criteria:


$$D_{i}=\{\begin{array}{c} 0,p_{i}^{*}\;<0\\ 1,p_{i}^{*}\;\geq 0 \end{array}\;\;\;$$ (1)

$$p_{i}^{*}=\gamma_{0}+\gamma_{1}X_{i}+\varepsilon_{i}$$ (2)

In Equations 1, 2, $p_{i}^{*}$ is the latent choice variable used to partition the treatment and control groups; $X_{i}$ is the observable variable, obtained by conducting Likelihood Ratio Test (LRT) on the variables listed in Table 2. Specifically, these variables include the teacher’s age, gender, job title, subject, whether they have contact with gifted students, and whether they are familiar with gifted education, as well as two interaction terms: the product of the teacher’s age and job title, and the product of whether teacher has contact with gifted students and whether the teacher is familiar with gifted education; $\varepsilon_{i}$ represents the unobserved heterogeneity of individuals in the choice process. The logit model is used to estimate the probability of teachers choosing to participate in gifted education professional development training.

**Table 2:** Reliability of the Instrument (*N* = 348).

**Variable**	**Item**	**Mean**	**SD**	**Item-test correlation**	**alpha**
Cognitive ability	cog1	2.72	0.849	0.8869	0.9683
	cog2	2.82	0.831	0.9179	0.9651
	cog3	2.70	0.858	0.9110	0.9660
	cog4	2.87	0.792	0.9272	0.9642
	cog5	2.84	0.817	0.9348	0.9634
	cog6	2.85	0.802	0.9422	0.9627
	cog7	2.89	0.827	0.9243	0.9644
	Test scale				0.9697
Interpersonal ability	inter1	2.73	0.870	0.8924	0.9605
	inter2	2.77	0.852	0.8822	0.9612
	inter3	2.60	0.945	0.8680	0.9632
	inter4	2.87	0.809	0.9055	0.9595
	inter5	2.82	0.819	0.9143	0.9589
	inter6	2.77	0.884	0.8858	0.9611
	inter7	2.75	0.847	0.9139	0.9589
	inter8	2.75	0.826	0.9214	0.9583
	Test scale				0.9650
Intrapersonal ability	intra1	2.76	0.849	0.9196	0.9793
	intra2	2.83	0.817	0.9388	0.9780
	intra3	2.74	0.827	0.9420	0.9778
	intra4	2.75	0.851	0.9430	0.9778
	intra5	2.79	0.840	0.9476	0.9775
	intra6	2.85	0.831	0.9444	0.9777
	intra7	2.88	0.837	0.9330	0.9784
	intra8	2.81	0.841	0.9422	0.9778
	Test scale				0.9807

Next, under the heterogeneity a hypothesis, the result model is as follows:


$$Y_{i}=\alpha +\beta D_{i}+\sum \gamma_{i}X_{i}+\varepsilon_{i}$$ (3)

In Equation 3, α represents the baseline value of student learning outcomes prior to the intervention; β denotes the heterogeneity of intervention effects, whose significance indicates whether participation in professional development training impacts student learning outcomes. This model partitions propensity scores into several intervals, using semiparametric or nonparametric methods to estimate the Average Treatment effect on the Treated (ATT) for treated individuals within each interval, then aggregates the results across intervals.

### 4.3 Sample selection

The target participants of this study consist of teachers from Chinese secondary schools (including junior high and senior schools) who are either involved in or responsible for the education and instruction of gifted students. The sample was primarily identified through two platforms:

The first is an explicit information source – the China Association for Science and Technology (CAST). This association is responsible for organizing and implementing China’s Middle School Talent Program, which has been in operation for over a decade since its launch in 2013. The program’s official website publicly lists the participating schools. Through collaborative research projects with the Association, we identified teachers, principals, and heads of teaching and research departments at relevant secondary schools; through these key intermediary figures, we further established contact with in-service secondary school teachers.

The second is an implicit source of information – tracing back to identify secondary school that offer the “Junior Class” by examining the student recruitment sources for the “Talent Program.” Specifically, the “Junior Class” is a distinctive program at the University of Science and Technology of China. Launched in 1978, it has been in operation for nearly half a century. Its admissions primarily select gifted and talented from secondary schools; some of these students have only recently entered junior high school but are admitted early to the university due to their outstanding performance for advanced training. Our research does not focus on these students who enter university early, but rather on using publicly available information about their high schools to compile a list of their former schools. We then use channels such as professional programs for teachers and research collaboration to contact with teachers or administrators at these high schools. This is because the fact that these students have the opportunity to enter university early indicates that their schools must have top-notch talent development programs in place to qualify for early university admission. It is precisely these secondary schools with such programs that we seek to identify.

Relying on the two official platforms mentioned above, we establish contact with current secondary school teachers through key intermediaries.

### 4.4 Sampling procedure

Data collection was conducted via an online platform (Questionnaire Star). During the sampling process, we mainly focused on two considerations: First, the similarity between the sample data and the actual regional distribution of the data. Teachers involved in gifted education constitute a sparse population, meaning their distribution across regions and schools is uneven. Taking into account the implementation of the Middle School Talent Program, the availability of regional resources, and program quality, we adopted a regional approach – covering the eastern, central, and western regions – to ensure that the sample distribution aligns as closely as possible with the actual distribution. Second, we included both teachers in gifted classes and teachers in mixed-ability classes, so as to contact as many as secondary schools as possible that implement the Middle School Talent Program or offer talent programs, minimizing omissions and ensure comprehensive sampling coverage.

Specifically, regarding regional characteristics, to ensure a more rigorous design, we stratified secondary school teachers based on regional distribution, with the sample ratio for the eastern, central, and western regions being approximately 5:3:2. Regarding school type, since Middle School Talent Program is a top-down policy implemented in public schools, our sample also focused on public schools to minimized selection bias.

Prior to data collection, in addition to briefly introducing the research content, we emphasized the following four points to teachers: First, participation is entirely voluntary. Since we contacted some teachers through department heads or principles, we stressed that their participation and responses would not be linked to their job performance, and that results would not be shared to administrators or authorities. Teachers were asked to answer based on actual circumstances to minimize social desirability bias. Second, inform that the survey is intended solely for academic research, and participants will have access to findings. Third, assure the confidentiality and anonymity of the dataset. Fourth, inform participants that they may withdraw at any time without penalty. Finally, explain that the estimated completion time is approximately 8–12 min. The ethics strictly adhere to the guidelines set forth in the Belmont Report.

### 4.5 Instrument and its reliability and validity

The instrument tool used is the Instructional Practice Questionnaire (IPQ) designed by Hong et al. (2011)^1^, which comprises three dimensions: cognitive, interpersonal and intrapersonal ability. The cognitive dimension includes seven items, such as “Students have opportunities to develop critical reading skills” and “Students have opportunities to apply problem-solving skills.” Interpersonal items in include eight questions such as “Students have opportunities to improve their relationships with their talented peers” and “Students have opportunities to practice active listening skills.” Intrapersonal measurement items include “Students have opportunities to set goals within areas of their interest” and “Students have opportunities to demonstrate a sense of responsibility,” among eight other items.

In the pilot study, the Chinese version of the questionnaire was reviewed by experts in gifted education and psychometrics to ensure content validity. A total of 80 pilot samples were collected, and the questionnaire content was revised according to the returned responses. For example, technical terms such as “procedural knowledge,” which frontline teachers might find difficult to understand, were removed. At the same time, an exploratory factor analysis was conducted on the pilot sample: the KMO statistic was 0.867, meeting the “good” standard, indicating the presence of common factors among the variables and confirming the suitability of factor analysis. The *p* value was < 0.001, rejecting the null hypothesis, that is, rejecting the assumption that the net correlations matrix is not an identity matrix and accepting the alternative hypothesis that the net correlation matrix is an identity matrix. This indicates the presence of common factors among the correlation matrices of the population, confirming the suitability of factor analysis. The reflectance matrix showed that the MSA values for all items ranged from 0.78 to 0.92, all exceeding 0.50, indicating that all items were suitable for factor analysis. The communality indices indicated that the communality of each item ranged from 0.61 to 0.83, all exceeding 0.20. Ultimately, there were three common factors with eigenvalues greater than 1, cumulatively explaining 71.81% of the total variance. Based on the rotated factor matrix, it is appropriate to name the three factors “Cognitive ability,” “Interpersonal ability” and “Intrapersonal ability,” respectively. Additionally, using the pilot sample, the clarity of the wording and the appropriateness of the language were verified, and the estimated completion time was determined to be less than 15 min.

In the formal survey, a total of 351 samples were collected. The collected data underwent the following checks: removal of excessive missing data, removal of data with all answers selected identically or with excessively short completion times, and removal of logically inconsistent responses. Ultimately, the number of valid samples was 348. The results of the reliability and validity tests for the formal survey are as follows.

As shown in the table above (Table 2), the Cronbach’s alpha coefficients for the three dimensions in the questionnaire range from 0.97 to 0.98, indicating that all dimensions exhibit “highly ideal” internal consistency. Furthermore, removing any single item would not improve the overall reliability of its respective dimension, suggesting that no item need to be deleted (Table 2).

In terms of validity (Table 3), the standardized factor loadings ranged from 0.82 to 0.95, indicating that each item effectively reflects its respective construct, and that the items are of high quality. The *R^2^* values ranged from 0.67 to 0.89, indicating that the three dimensions provide good explanatory power for the items they comprise. The Average Variance Extracted (AVE) values were all 0.8 or higher, indicating that the three dimensions possess excellent convergent validity. Since the instrument is adapted from another well-established questionnaire, we had expected its internal consistency and reliability to be sufficiently good.

**Table 3:** Validity of the instrument (*N* = 348).

**Variable**	**Item**	**λ**	***R*** ^**2**^	**CR**	**AVE**
Cognitive ability	cog1	0.850	0.723	0.970	0.822
	cog2	0.890	0.791		
	cog3	0.882	0.779		
	cog4	0.921	0.848		
	cog5	0.933	0.870		
	cog6	0.945	0.893		
	cog7	0.923	0.852		
Interpersonal ability	inter1	0.855	0.732	0.965	0.777
	inter2	0.843	0.710		
	inter3	0.818	0.669		
	inter4	0.908	0.825		
	inter5	0.915	0.837		
	inter6	0.880	0.775		
	inter7	0.911	0.830		
	inter8	0.916	0.840		
Intrapersonal ability	intra1	0.903	0.816	0.981	0.865
	intra2	0.930	0.864		
	intra3	0.933	0.871		
	intra4	0.933	0.870		
	intra5	0.942	0.887		
	intra6	0.937	0.878		
	intra7	0.925	0.856		
	intra8	0.935	0.875		

## 5 Findings

### 5.1 Sample characteristics

During the formal survey phase, we received responses from secondary school teachers in 14 cities across 9 provinces in eastern, central, and western China, resulting a total of 348 valid responses.

As shown in Table 4, the sample spans ages 22 to 60, covering all age groups of in-service teachers. Furthermore, the subjects taught by these teachers encompass all disciplines at the secondary school level, thus ensuring considerable representativeness.

**Table 4:** Sample information (*N* = 348).

**Variable**	**Number**	**Percent**
Age	22 (Min) 60(Max)	38.78 (Mean) 8.96 (SD)
Years of teaching	0 (Min) 40(Max)	14.49 (Mean) 9.98 (SD)
Gender		
Female	184	52.87%
Male	164	47.13%
Job position		
Instructor	321	92.24%
Manager with teaching responsibility	21	6.03%
Administrator	6	1.72%
Subject		
Language	44	12.64%
Math	53	15.23%
Foreign language	42	12.07%
Moral Education	25	7.18%
History	17	4.89%
Geography	23	6.61%
Physics	35	10.06%
Chemistry	36	10.34%
Biology	26	7.47%
Computer	16	4.60%
Physical Education	16	4.60%
Arts	5	1.44%
Others	3	0.86%
Missing data	7	2.01%
Contact with G&T students		
No	137	39.37%
Yes	211	60.63%
Knowledge about G&T education		
No	147	42.24%
Yes	201	57.76%
Grade		
7th grade	40	11.49%
8th grade	30	8.62%
9th grade	53	15.23%
Mixed junior high school grades	8	2.30%
10th grade	132	37.93%
11th grade	30	8.62%
12th grade	45	12.93%
Mixed senior high school grades	8	2.30%
Teacher’s self-assessment		
Normal	281	80.75%
Gifted and Talented	67	19.25%
Teacher’s education background		
Associate	23	6.61%
Bachelor	209	60.06%
Master	109	31.32%
Doctoral	3	0.86%
Missing data	4	1.15%
Teacher’s parents’ education background		
Associate	72	20.69%
Bachelor	33	9.48%
Master	1	0.29%
Doctoral	2	0.57%
Missing data	240	68.97%
School location		
Rural	30	8.62%
Small town	22	6.32%
Suburb	66	18.97%
Urban area	155	44.54%
Downtown	75	21.55%
School level		
Junior high only	114	32.76%
Senior high only	181	52.01%
Both junior and senior	53	15.23%
Total number of students at the school	2,695 (Median)	1,500 (25th percentile) 4,015 (75th percentile)

In addition, participants were asked to provide the names of the G&T programs at their schools, including “Shing-Tung Yau Youth Class,” “Pure Innovation Class,” “Olympic School,” and “Youth Engineering Institute,” etc. This information also serves as evidence of the validity of the data.

Among the variables related to sample characteristics listed above, the following were identified through LRT test as suitable matching variables: teacher’s age, gender, job title, subject, contact with G&T students, and knowledge about G&T students.

### 5.2 Matching effect

The balancing test results in Table 5 show: First, the absolute values of standardized differences after matching remained below 10%. Second, the bias in nearly all covariates significantly decreased, indicating that matching improved balance. Third, covariates with significant differences before matching (*p* < 0.01, *p* < 0.1) became non-significant after matching, suggesting that matching reduced differences in means. Fourth, all variables exhibiting excessive intergroup differences showed improved performance after matching. Finally, summary metrics reveal that the post-matching mean bias distance falls well below the 25% critical threshold, while variance differences narrow to an acceptable range, with overall effectiveness significantly enhanced. Variables within the model achieved the intended outcomes.

**Table 5:** Balance test.

**Variable**	**Unmatched**	**Mean**		**%bias**	**%reduct**	**t-test**		**V(T)/**
	**Matched**	**Treated**	**Control**		**|bias|**	***t***	***p* > *t***	**V(C)**
Age	U	37.28	38.31	−11.4	73.4	−1.02	0.309	0.84
	M	37.19	37.46	−3.0		−0.33	0.743	1.03
Gender	U	1.50	1.42	16.9	49.0	1.49	0.136	1.02
	M	1.47	1.51	−8.6		−0.85	0.394	1.00
Position	U	1.11	1.05	20.8	88.3	1.72	0.086	3.13*
	M	1.04	1.04	−2.4		−0.36	0.716	1.08
Subject	U	8.95	9.08	−4.0	−143.4	−0.35	0.723	1.05
	M	8.97	8.66	9.8		0.96	0.339	1.00
Direct contact	U	1.72	1.43	61.2	99.6	5.50	0.000	0.83
	M	1.69	1.69	−0.3		−0.03	0.978	1.00
Indirect knowledge	U	1.71	1.36	75.1	96.1	6.71	0.000	0.89
	M	1.69	1.71	−3.0		−0.30	0.765	1.03
Age*position	U	42.11	39.96	13.1	75.5	1.08	0.280	3.19*
	M	38.44	38.97	−3.2		−0.48	0.629	1.10
Direct*indirect	U	3.05	2.01	86.2	98.5	7.54	0.000	1.18
	M	2.96	2.98	−1.3		−0.12	0.905	1.03

**Sample**	**Ps *R*2**	**LR chi2**	***p* > chi2**	**MeanBias**	**MedBias**	***B***	***R***	**%Var**
Unmatched	0.141	62.83	0.000	36.1	18.9	87.9**	2.50**	25
Matched	0.004	2.19	0.975	3.9	3	14.9	1.32	0

* If variance ratio outside [0.77; 1.30] for U and [0.76; 1.32] for M. ** If B > 25%, R outside [0.5; 2].

**Figure 2:**
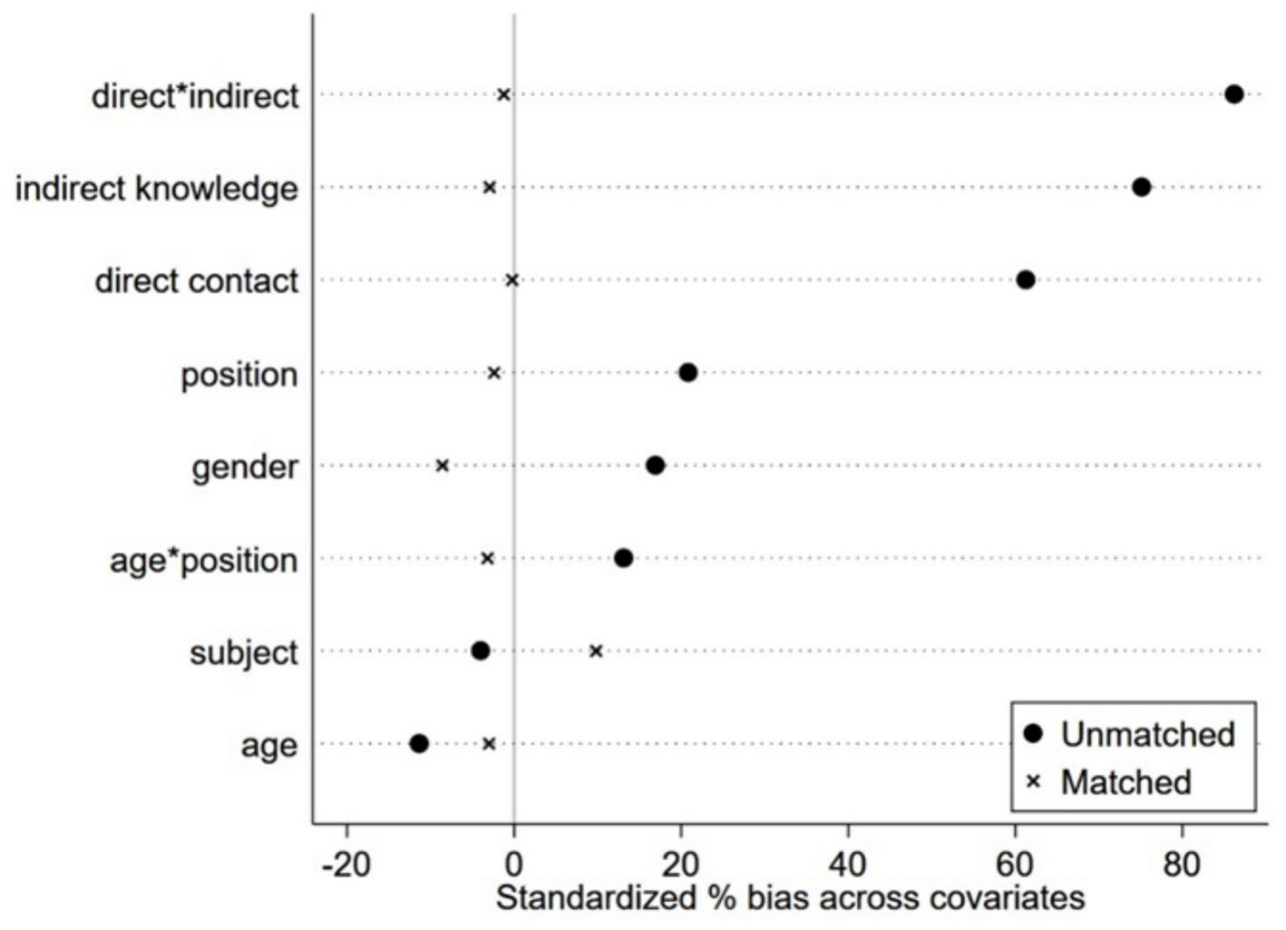
Balance test.

**Figure 3:**
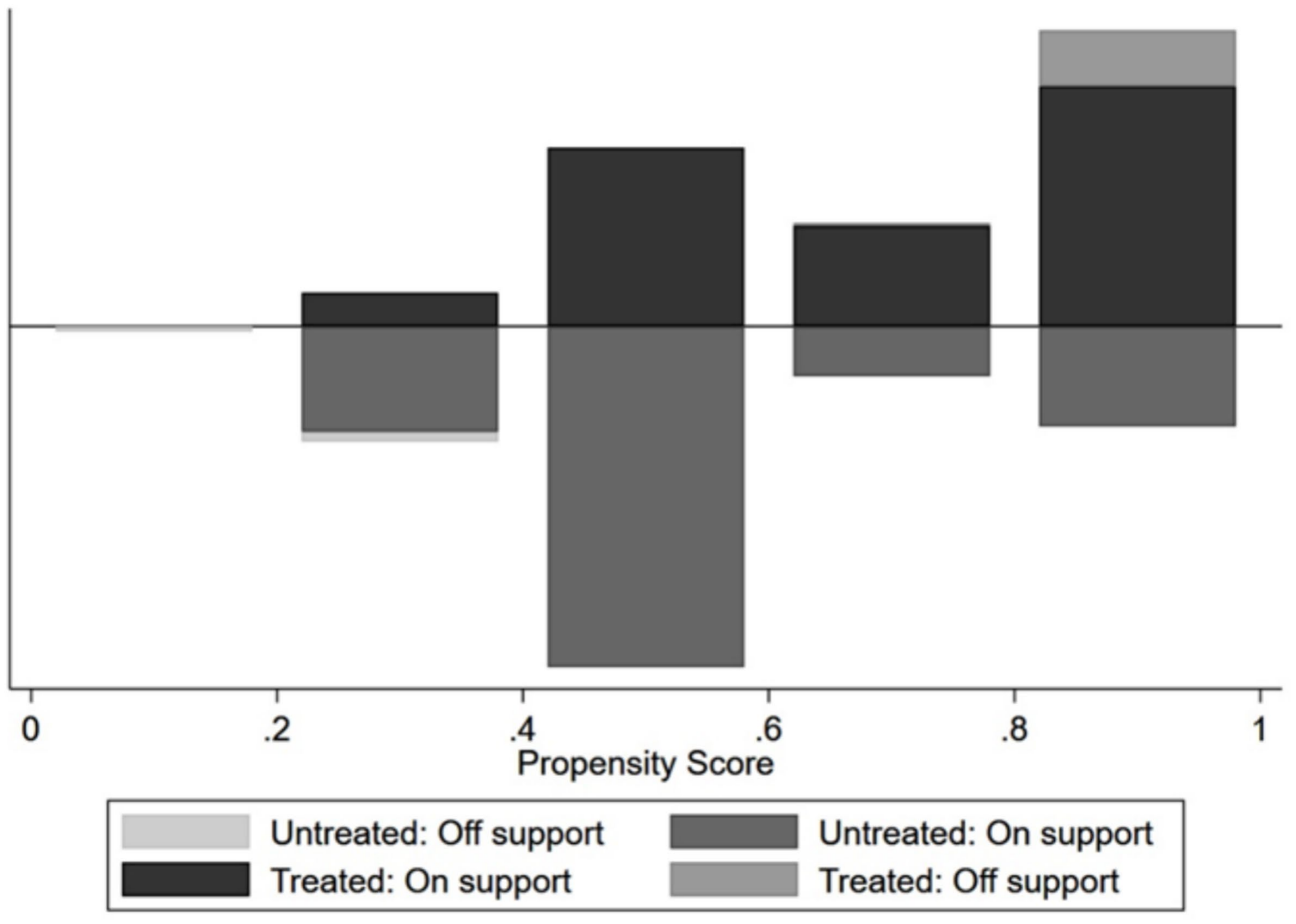
Common Support Test.

In terms of balance, the standardized deviations of covariates prior to matching were relatively large, with some exceeding 80%. Meanwhile, after matching, the covariate deviations between the treatment and control groups approached 0 (see Figure 2). Regarding joint support, a significant number of samples in the control group did not overlap within the joint support range, and the same was true for the treatment group. A close correspondence exists between the samples of the two groups (see Figure 3).

### 5.3 Propensity score matching, stratification, and weighting

We employed six estimation methods based on semi-parametric and non-parametric approaches: nearest neighbor matching, kernel matching, local linear regression matching, spline matching, Mahalanobis distance matching, and stratified matching (see Table 6). These yielded the Average ATT for student learning gains from the intervention.

**Table 6:** Gifted education PD enhance students’ 21st century skills but exhibit heterogeneity.

Matching	1–4 nearest neighbors with callipers	Kernel	Local linear regression	Spline	Mahalanobis distance	Propensity score stratification	Propensity score weighting
	(Bootstrap Std. Err.)				(Std. Err.)		(Robust Std. Err.)
ATT	0.243*	0.279**	0.228**	0.255**	0.301***	0.266***	0.236**
	(0.132)	(0.115)	(0.103)	(0.110)	(0.107)	(0. 109)	(0.109)
ATU	0.276**	0.285***	0.301***	0.285***	0.329***	–	–
	(0.134)	(0.099)	(0.085)	(0.079)	(0.101)	–	–
ATE	0.255**	0.281***	0.255***	0.266***	0.311***	–	0.257***
	(0.114)	(0.100)	(0.090)	(0.089)	(0.104)	–	(0.095)
Obs	341					338	341

***p < 0.01, **p < 0.05, *p < 0.1.

As shown in Table 6, the estimated ATT results for nearest neighbor matching, kernel matching, local linear regression matching, spline matching, Mahalanobis distance matching, and hierarchical matching are 0.243, 0.279, 0.228, 0.255, 0.301, and 0.266, respectively, indicating relatively close values.

To compare changes in teaching practices under the same standard, we further employed propensity score weighting. Propensity score weighting differs from the other six matching methods mentioned above in that the latter six use distinct approaches to estimate distances between samples. Propensity score weighting, however, converts propensity scores into weights using a specific formula and employs OLS to estimate the ATT. The weight calculation formula is as follows:


$$\left\{ \begin{array}{c} \mathrm{ATT}\_\omega =p/(1-p)\;\;\;\;(D_{i}=0)\\ \mathrm{ATT}\_\omega =1\;\;\;\;(D_{i}=1) \end{array}\;\right.$$ (4)

$$\left\{ \begin{array}{c} \mathrm{ATU}\_\omega =1\;\;\;(D_{i}=0)\\ \mathrm{ATU}\_\omega =(1-p)/p\;\;\;\;(D_{i}=1) \end{array}\right.$$ (5)

$$\left\{ \begin{array}{c} \mathrm{ATE}\_\omega =1/(1-p)\;\;\;\;(D_{i}=0)\\ \mathrm{ATE}\_\omega =1/p\;\;(D_{i}=1) \end{array}\right.$$ (6)

The weighting results of propensity scores calculated using Equations 4–6 are presented in the final column of Table 6. Furthermore, by comparing the ATT and ATU values in Table 6, it can be observed that, overall, the positive impact of teacher participation in PD training on student learning outcomes is smaller than the positive effect observed when teachers did not receive such training. This suggests that the heterogeneity hypothesis may be supported.

### 5.4 Heterogeneous treatment effects

**Figure 4:**
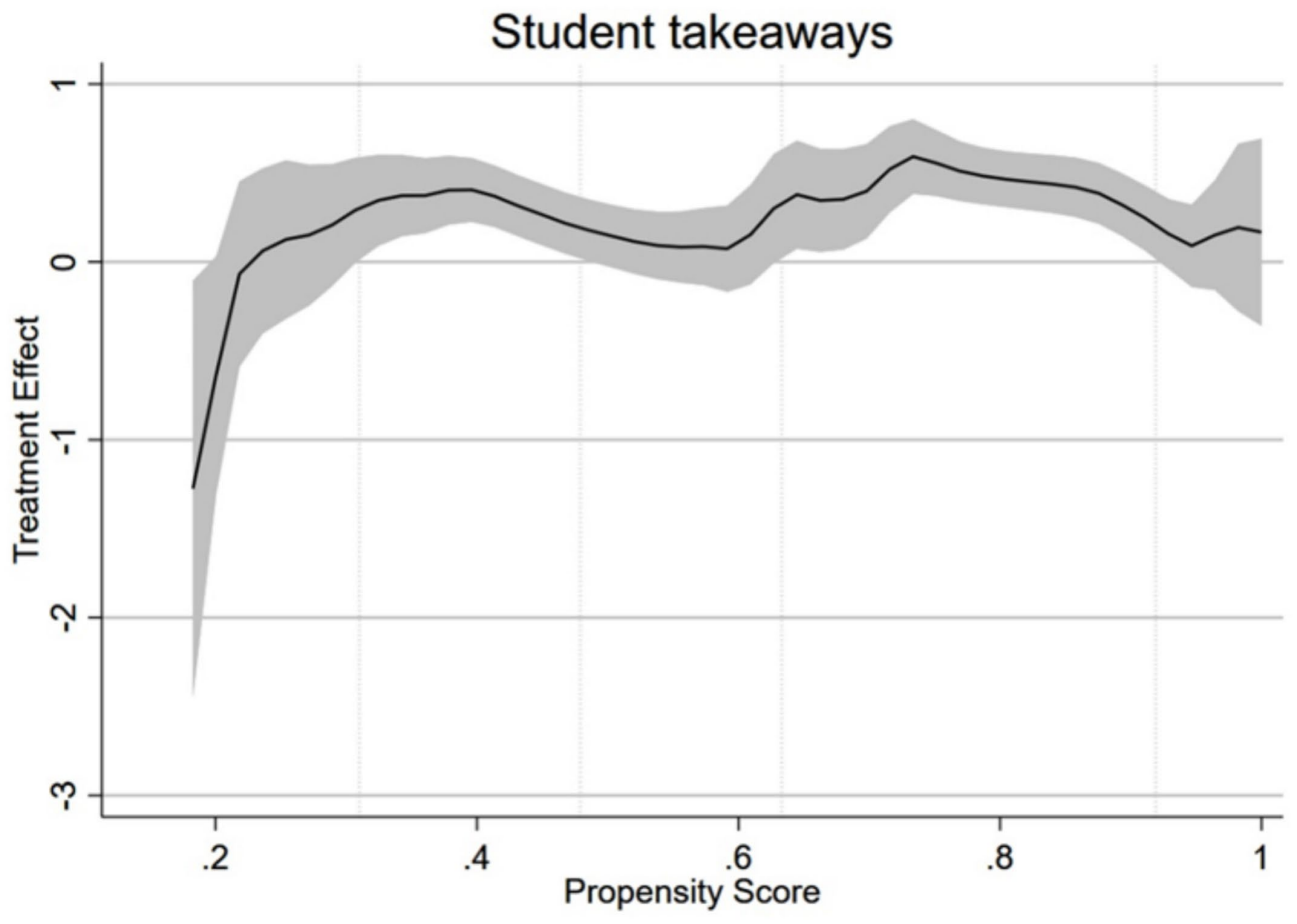
The M-shaped heterogeneous impact of gifted education PD on student outcomes.

Heterogeneous Treatment Effects (HTE) refers to the possibility that the impact of teacher participation in PD training on student learning outcomes may vary depending on the teacher’s career stage. Figure 4 presents the HTE results for student achievement following specialized training in gifted education.

In Figure 4, the horizontal axis represents the probability of participants enrolling in gifted education training programs; the vertical axis indicates the likelihood of training yielding positive outcomes for students; the solid black line denotes the average change rate corresponding to each propensity score; the shaded area represents the confidence interval (CI). If the CI includes the value 0, the null hypothesis (H0: ATE = 0) is unacceptable at the 95% confidence level, meaning student outcomes show no change. Conversely, if the CI excludes 0, it indicates a significant change in student learning outcomes.

As previously mentioned, teachers’ willingness to participate in gifted education training is influenced by their direct contact with gifted students or their understanding of gifted education. Therefore, the propensity score is affected by teachers’ relevant experience in the field of gifted education. In other words, the moderating effect manifests as an interaction: teacher age and the probability of teachers choosing to participate in training jointly influence student learning outcomes, but this relationship is not linear. As shown in Figure 4, the impact of teacher participation in professional training on student learning gains exhibits an overall “M”-shaped pattern. Based on whether the gray shaded confidence intervals include zero, the trend of the black line can be broadly divided into five stages, aligning precisely with the five career stages of teachers as categorized by Huberman. During the second and fourth career stages, teacher participation in PD significantly enhances student learning outcomes. Conversely, during the first, third, and fifth stages, students’ cognitive, interpersonal, and intrapersonal skills do not appear to show marked improvement following teacher participation in gifted education training.

### 5.5 Robustness check

To conduct robustness check, we used the Rosenbaum method. Results indicate that ATT is only affected when a highly significant unobserved factor exists, doubling the likelihood of teacher participation in professional development (*p* < 0.01). Thus, the findings demonstrate high robustness.

## 6 Conclusion and discussion

The education field has long emphasized personalized instruction tailored to individual needs, and teacher training is no exception. Over the past 25 years, scholarly efforts to focus on the core characteristics of effective professional development have grown rapidly, leading to a proliferation of concise design checklists. However, such apparent consensus can easily lead to the misconception that these conclusions are based on rigorous evidence, when in fact their empirical foundation is problematic (Asterhan and Lefstein, 2024). This study aims to address this gap. UNESCO (2019) stipulates that professional learning should be an indispensable component of every teacher’s career, spanning their entire professional journey and possessing a global perspective. Recently, the Chinese government has actively response by initiating policies to promote PD training for all teachers. Based on this, we extended the PCK theoretical model to the field of gifted education to examine the effectiveness of teacher professional development. Using a sample of in-service secondary school teachers, this empirical study evaluates the impact of professional development training on student gains, and provide evidence for the personalized design of such training. Propensity Score Matching (PSM) and Heterogeneity of Treatment Effect (HTE) models are used to address endogeneity issues such as omitted important variables and sample self-selection.

Overall, contrary to the common belief that “the more experienced a teacher is, the better their teaching outcomes,” this study found that the impact of teacher experience on learning outcomes shows a “bimodal” pattern. At the two peaks of this “M”-shaped curve, specifically, during the 4th to 6th, 19th to 30th years of teaching, participation in PD about gifted education significantly promotes the development of these talent students.

In detail, joining in professional development has led to noticeable improvements in the development of teachers who are in the Career Stability Phase (Stage 2) and the Career Serenity/Conservation Phase (Stage 4). According to Huberman’s classification of teachers’ life cycle, teacher in the Stability Phase (Stage 2) have completed their period of practical adaptation and are beginning to feel excited about exploring new things. They have accumulated sufficient foundational classroom experience while retaining the exploratory spirit of early-career teachers who have not yet been constrained by daily routines. These factors combined make the career stability phase a critical window for translating the effectiveness of PD in gifted education. Similarly, teachers in the Career Serenity/Conservation Phase (Stage 4, with 19–30 years of experience) also exert a significant impact on student growth following participation in PD about gifted education. Sims et al. (2023) proposed an IGMP framework for professional development, arguing that motivation is the most critical, and the most easily overlooked component in the conversion of PD outcomes. If professional development programs lack incentive mechanisms aligned with teacher’s intrinsic motivation, the acquisition of Pedagogical Knowledge (PK) and Content Knowledge (CK) will not automatically result in practical changes. This aligns with Huberman’s description of teachers in the Serenity/Conservation phase of their careers. He notes that the 4th stage of life cycle is a restructuring phase that shifts from being driven by external recognition to the pursuit of intrinsic meaning. Teachers in Serenity/Conservation phase no longer seek validation through external evaluations but actively pursue professional challenges with intrinsic significance. This internal motivation makes the transfer of knowledge into practice particularly thorough, ultimately yielding a significant impact on the development of gifted students.

However, professional development in gifted education have not empowered teachers in Stage 1, 3, and 5, that is, for students taught by teachers in these stages, student outcomes have not shown marked improvement following teachers’ participation in PD.

With regard to teachers in the first stage of their careers, Admiraal et al. (2023), drawing on cross-nationals date from 24 countries, found that newly hired teachers generally feel that are not adequate prepared for the job. Nevertheless, PD programs for gifted education require teachers to simultaneously master pedagogical knowledge and content knowledge, while also responding to the unique social–emotional needs of gifted students. Particularly when workloads are excessive, or when the content of PD programs is too far from the immediate survival needs of novice teachers, such programs may actually become an additional source of stress rather than a supportive resource. The most direct implication for PD in gifted education is that investing in specialized PD training about gifted education for novice teachers who are in the entry and adaptation phases is a suboptimal choice in terms of efficiency.

For teacher in the third stage (with 7–18 years of experience), Huberman describes this phase as a period of either active growth or self-doubt – some teachers successfully enter a new cycle of career advancement, while others fall into crisis or burnout. According to Sims et al. (2023), since knowledge alone is unlikely to bring about changes in practice. Therefore, professional fail to yield educational outcomes if it does not motivate teachers to adopt goals around changing their practice. The findings of this study also confirm the high heterogeneity of teachers’ motivational states at this stage (Farias et al., 2012): for some teachers in a state of self-doubt, PD occur during a window of motivational disengagement, where the acquisition of new knowledge clashes sharply with the reconstruction of job meaning. This situation, combined with that of other teachers in a proactive state, systematically offsets the impact of PD programs overall.

With regard to teacher nearing the end of their careers, Brouhier et al. (2023) argue that their work resources and demands are in conflict with one another. This is because the field of education is characterized by the constant updating and evolution of knowledge; students’ heterogeneity, technological innovations, and the implementation of educational reform policies all place ongoing pressure on teachers nearing the end of their careers. The rapidly changing and increasingly demanding external environment undermines the health and work quality of these teachers.

## 7 Implications and limitations

The main finding of this study is that professional development related to gifted education has a significant conditional impact on students’ cognitive, interpersonal and intrapersonal development, with the nature of this impact varying depending on the stage of the teacher’s life cycle. This provides both theoretical and practical insights for programs targeting gifted students and teacher professional development:

In terms of theoretical contributions, first, this study responds to the calls that have merged over the past decade or so to address the unique developmental needs of gifted students. For nearly a century, beginning with Lewis Terman, discussions about gifted students have focused solely on static outcomes in the single dimension of cognitive ability, with their non-cognitive or socio-emotional competencies were not only neglected but even deemed unnecessary. The root cause lies in teachers’ lack of professional understanding of the multidimension developmental needs of gifted students, which is also the core reason for the failure of differentiated instruction. Baccassino and Pinnelli (2023) use the term “myth of happiness” to describe a common misconception among teachers regarding gifted students. This refers to teachers’ neglect of the sentimental complexity of gifted students and their mistaken belief that such students are always happy. The misconception like this obscures the genuine social–emotional and relational complexities present in gifted students. This study provides an empirical foundation for the 21st Century Skills framework to serve as a tool for evaluating the effectiveness of PD.

Furthermore, this study advances the theory of teacher professional development stages from a descriptive framework of characteristics to a predictive, explanatory framework. A meta-analysis by scholars from Harvard University and Brown University, integrating 60 causal studies, note that effective professional development is individualized, time-intensive, sustained, context-specific, and focused on discrete skills (Kraft et al., 2018). These studies attribute the effectiveness of PD to the design of the institutional environment, while the role of teachers remains ambiguous. In contrast, based on Huberman’s classification of teacher life cycles, this study extends the theoretical significance from describing the characteristics of PD to explaining how teachers’ psychological states moderate the transfer of PD program effects.

Regrading teaching practice, this study found that the effectiveness of professional development programs for gifted education teachers depends not only on the program design itself but also, to a large extent, on the degree of fit with the teachers as recipients: prioritizing the allocation of resource-intensive gifted education programs to teachers in the second (stabilization) and fourth (serenity/conservation) stages of their careers may enable the precise allocation of limited resources to high-efficiency recipients, thereby enhancing the overall return on investment in gifted education PD programs. As for teachers in the first stage (entry) of their careers, preparatory training should be design with gifted education as the core objective, rather than focusing on a comprehensive specialized knowledge system; for teachers in the third stage (active/self-doubt) of their careers, particularly those experiencing self-doubt, support should first be provided to help them redefine their professional purpose, followed by the gradual introduction of content related to gifted education; for teachers in the fifth stage (disengagement) of their careers, evaluations of PD programs should better distinguish between “impact on students” and “values to teachers”. Providing teachers in the final stage with more opportunities to participate in PD may enhance their professional well-being. In this way, this study established a comprehensive strategy for improving the efficient of resource allocation across teacher’s life cycle.

Like all educational improvement models, this study faces certain insurmountable challenges. Although the causal inference within the model is grounded on a theoretical framework, more research could conduct measurements at different time points for further validity. Additionally, the findings in this study are primarily applicable to the secondary school level and require extension to other educational stages to verify. Furthermore, since the professional development of teachers in this study focused solely on the field of gifted education, its applicability to general education or other special education settings is limited.

## Data Availability

The raw data supporting the conclusions of this article will be made available by the authors, without undue reservation.

## Footnotes

1 Reproduced with permission from the author, Hong et al. (2006).
